# One-Lung Ventilation in a Patient With Necrotizing Pneumonia Complicated by a Bronchopleural Fistula

**DOI:** 10.7759/cureus.15138

**Published:** 2021-05-20

**Authors:** Kellen T Creech, Umar Chaudhry

**Affiliations:** 1 Internal Medicine, Dr. Kiran C. Patel College of Osteopathic Medicine at Nova Southeastern University, Davie, USA; 2 Internal Medicine, Westside Regional Medical Center, Plantation, USA

**Keywords:** necrotizing pneumonia, one lung ventilation, one-lung isolation, protective ventilation, thoracic anesthesiology

## Abstract

A 32-year-old male with morbid obesity presented to the emergency department with a one-week history of shortness of breath and productive cough. This patient had previously been evaluated at an urgent care facility, diagnosed with pneumonia, and prescribed oral antibiotics. This patient’s worsening shortness of breath and productive cough led this patient to seek further care at the emergency department. Chest radiography revealed acute respiratory distress syndrome (ARDS) with an empyema in the right pleural space. He was admitted to the intensive care unit and subsequently intubated due to severely compromised cardiopulmonary function. The patient then underwent irrigation of the chest cavity and chest tube placement for drainage of the right-sided empyema. Surgical cultures revealed growth of Streptococcus anginosus and appropriate antibiotics were started. The patient’s pulmonary function continued to deteriorate and this patient was placed on venous to venous extracorporeal membrane oxygenation (VV ECMO). Due to continued respiratory failure and a persistent air leak, a double-lumen endotracheal tube (DLT) was exchanged to initiate one-lung ventilation (OLV) to optimize ventilation and protect the lung containing the empyema. Over the following days, worsening leukocytosis and atelectasis were noted upon imaging prompting cardiothoracic surgery to return to the operating room to perform a right posterolateral thoracotomy and full right lung decortication. The procedure was successful and a bronchopleural fistula (BPF) was observed, secondary to the necrotizing pneumonia. The observation of the fistula explained the persistent air leak and issues maintaining adequate oxygenation. OLV through a DLT was continued over the following days, and the patient’s pulmonary status and leukocytosis ultimately began to improve over the next two weeks. This patient was then able to be weaned off the EMCO device and was extubated. The patient was stabilized and discharged to a rehabilitation facility for further recovery. This case highlights how the use of lung isolation techniques were essential in the recovery of this patient with an estimated 50% mortality rate due to significant pulmonary injury from necrotizing pneumonia and complicated by a BPF.

## Introduction

In most patients requiring mechanical ventilation, both lungs are inflated and deflated in synchrony through a single lumen endotracheal (ET) tube. In certain circumstances, lung isolation techniques, including one-lung ventilation (OLV), may be indicated to facilitate intrathoracic surgical field exposure, or less commonly to prevent damage to the contralateral lung when an abscess or pulmonary hemorrhage is present, or avoid ventilation of a unilateral bronchopleural fistula (BPF) or bullae [1]. A comprehensive understanding of tracheobronchial anatomy and cardiopulmonary physiology are essential for successful single lung isolation [2]. One trial revealed that 39% of anesthesiologists with limited thoracic experience were unable to successfully achieve lung isolation due to unfamiliarity of the endoscopic anatomical view of the airway [2]. Furthermore, malpositioning of the double-lumen endotracheal tube (DLT) is the most common cause of intraoperative hypoxemia during OLV [2]. BPFs are indications for lung isolation techniques and are significant causes of mortality and morbidity due to their ability to significantly compromise cardiopulmonary function [2]. Lung protective strategies, surgical intervention, and appropriate antibiotic coverage are key in the treatment of BPFs and patients with severely comprised pulmonary function. Components of these lung-protective strategies include low tidal volumes calculated at 4 to 8 mL/kg of predicted body weight, peak plateau pressures (pPlat) less than 30 cm H2O, permissive hypercapnia, and 5-10 cm H2O of positive end-expiratory pressure (PEEP) [3]. We present the case of a patient with a one-week history of shortness of breath and productive cough, upon which further imaging revealed acute respiratory distress syndrome (ARDS) secondary to necrotizing pneumonia and furthermore complicated by a BPF, where lung isolation techniques were utilized in the recovery of this patient over a 33-day hospital stay.

## Case presentation

**Figure 1:**
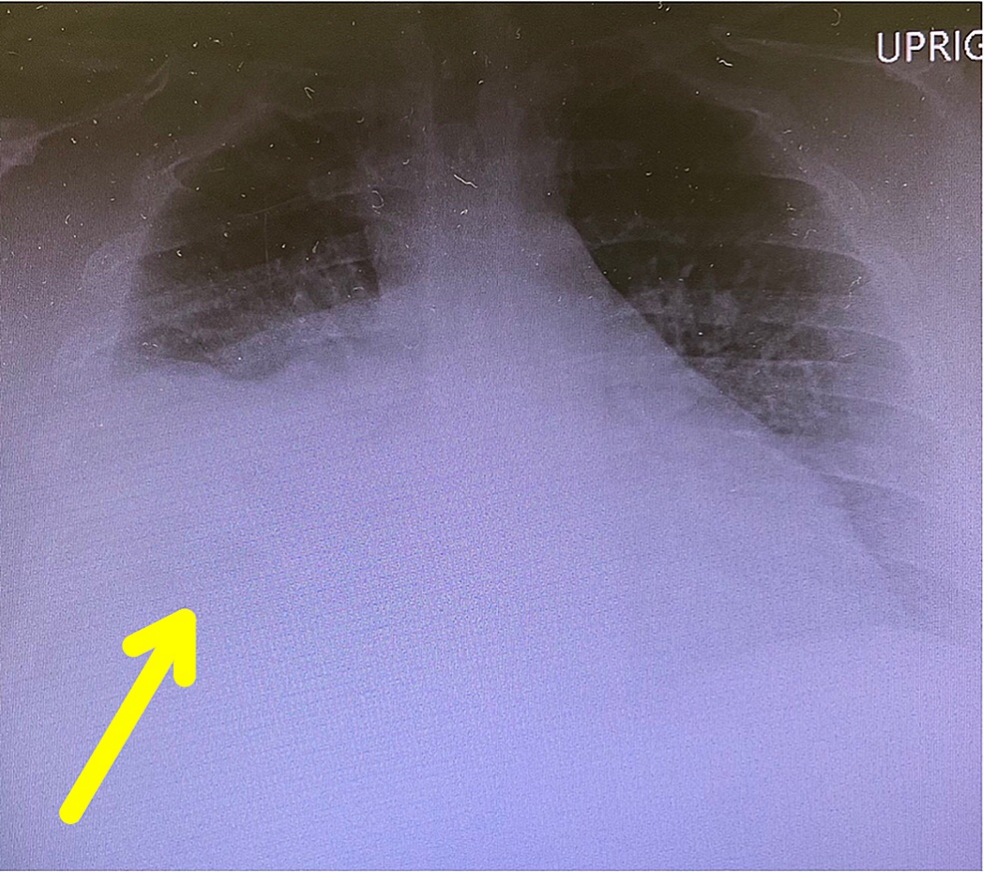
Chest radiograph from the initial day of presentation revealing a small to moderate right pleural effusion with associated consolidation (yellow arrow)

**Figure 2:**
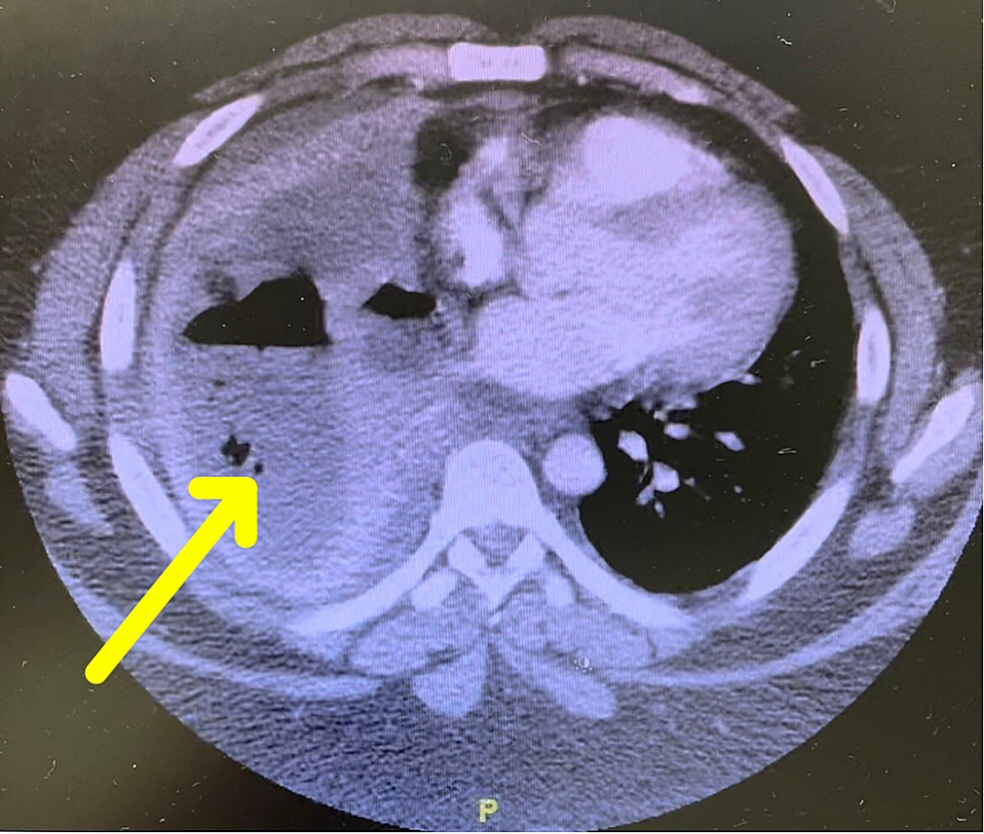
Computed tomography (CT) of the chest from the initial day of presentation revealing a 12.2 cm x 12.1 cm loculated gas and fluid collection within the right lower lobe concerning for pulmonary abscess vs empyema (yellow arrow)

A 32-year-old obese male with no prior medical history presented to the emergency department with a one-week history of shortness of breath and productive cough. Initial imaging revealed significant diffuse opacities consistent with ARDS and a substantial empyema was noted in the right lower lobe (Figures 1-2).

Other initial notable laboratory results included significant leukocytosis (white blood cells: 31.3 10^9/L), anemia (hemoglobin: 12.1 g/dL), D-Dimer of 3181 ng/mL, and arterial blood gas (ABG) revealed the patient to be in acute hypoxic respiratory failure. The patient was provided respiratory support through a non-rebreather mask initially, but subsequently required intubation and respiratory support through a ventilator and was transferred to the intensive care unit.

Considering this patients’ diagnosis of ARDS, the ARDSNet guidelines were employed. This consisted of lung-protective strategies including ventilation settings of tidal volumes calculated at 6 mL/kg of ideal body weight, a respiratory rate of 26 breaths per minute, a tidal volume of 460 mL, positive end-expiratory pressure (PEEP) of 14 cmH2O, and maintaining an oxygenation saturation greater than 90%. In addition to this, sedation and paralysis were initiated intravenously, inhaled nitrous oxide was initiated at 20 parts per million, and the steroid Decadron was given at 6 milligrams intravenously every 24 hours for 7-10 days. Proning would normally be indicated in patients with ARDS, but in this patient was unable to be performed due to his obese body habitus and resulting difficulties in access to the chest tubes. A persistent air leak was observed after intubation, which was attributed to a possible complication of the necrotizing pneumonia present in the lower lobe of the right lung. Shortly after arrival to the intensive care unit, bronchoscopy was performed. Bronchoscopy revealed thick, mucous plugs, purulent secretions, friable mucosa, and significant erythema. Bronchoalveolar lavage was also performed at that time. Cultures were obtained during the bronchoscopy that revealed the growth of Streptococcus anginosus and appropriate antibiotic coverage was initiated.

**Figure 3:**
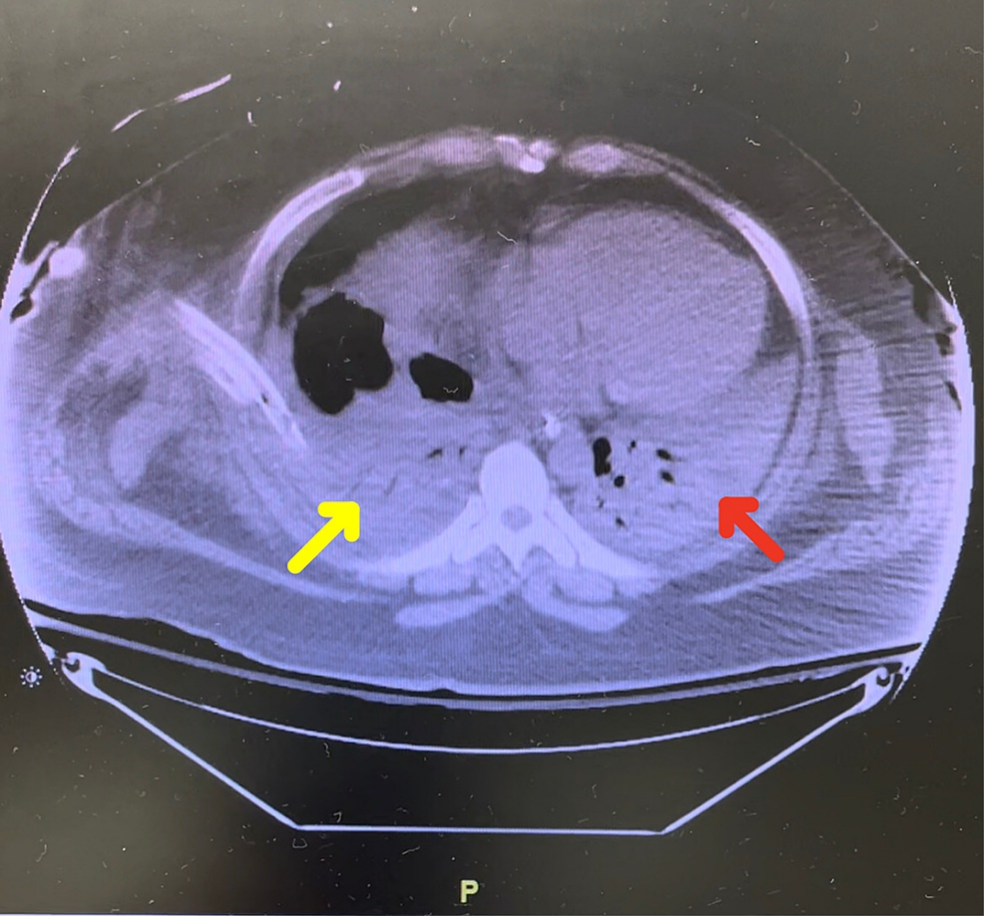
CT of the chest from day 8/33 of hospitalization showing complete atelectatic collapse of the left lung (red arrow) and right lung and a loculated right basilar hydropneumothorax (yellow arrow)

The following day, the patient was taken to the operating room for surgical evacuation of the empyema, irrigation of the chest cavity, and the placement of two chest tubes for residual drainage of the empyema. However, over the following days, this patient continued to remain acidemic, hypercarbic, and hypoxemic despite the use of airway pressure release ventilation (APRV) and inhaled nitrous oxide. To measure the extent of lung injury, the Murray score of this patient was calculated to be 3.8. Extracorporeal membrane oxygenation (ECMO) was then discussed and agreed upon with the patient’s family. This patient tolerated the cannulation procedure well and the ventilation settings were adjusted to a tidal volume of 320 mL (4 mL/kg), PEEP of 12 cm H2O, fraction of inspired oxygen 85%, maintaining the patient’s pH at or above 7.2 and to maintain the partial thromboplastin time between 50-70 seconds. These settings were in accordance with the Extracorporeal Life Support Organization (ELSO) guidelines. Given the inadequate tidal volume on the pressure control ventilator mode, due to the persistent air leak from the right chest tube, the ventilator mode was changed to assist control. Follow-up imaging on day 8 of 33 revealed left lung atelectasis and continued right lobar consolidation (Figure 3).

**Figure 4:**
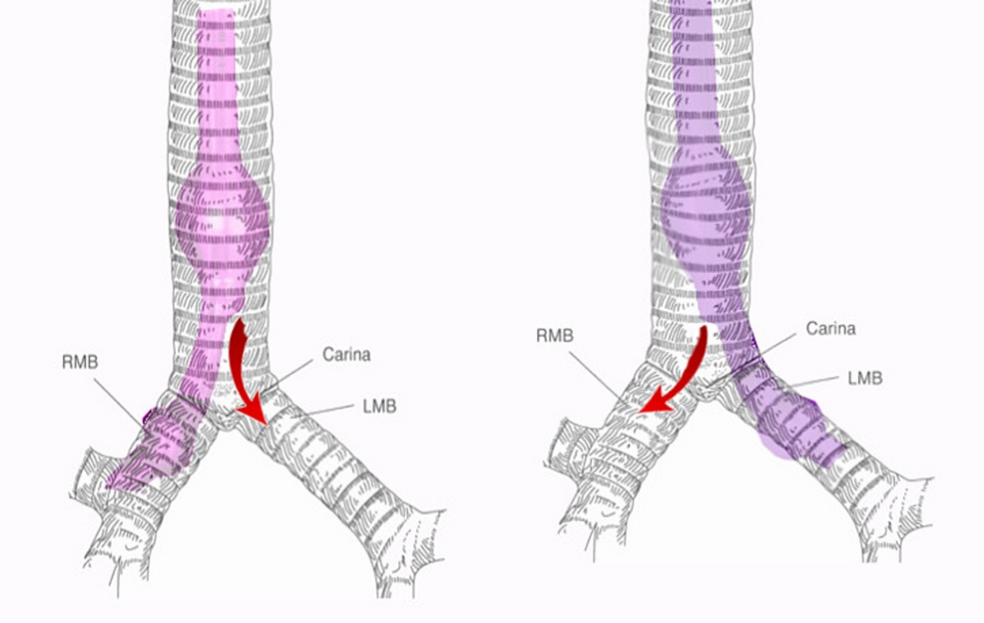
Illustration of a right double-lumen endotracheal tube (DLT) (left) and a left DLT (right) and correct placement within the tracheobronchial tree. The red arrows signify the movement of air from the tracheal lumen of the DLT [4]

Due to the worsening atelectasis and continued acidemia and hypercarbia, it was decided to replace the single lumen ET tube with a left DLT. An illustration of left and right DLT with correct placement within the tracheobronchial tree is seen in Figure 4 below [4]. The patient tolerated the procedure well and visualization of the bronchial lumen of the left DLT extending into the left mainstem bronchus was confirmed via bronchoscopy.

**Figure 5:**
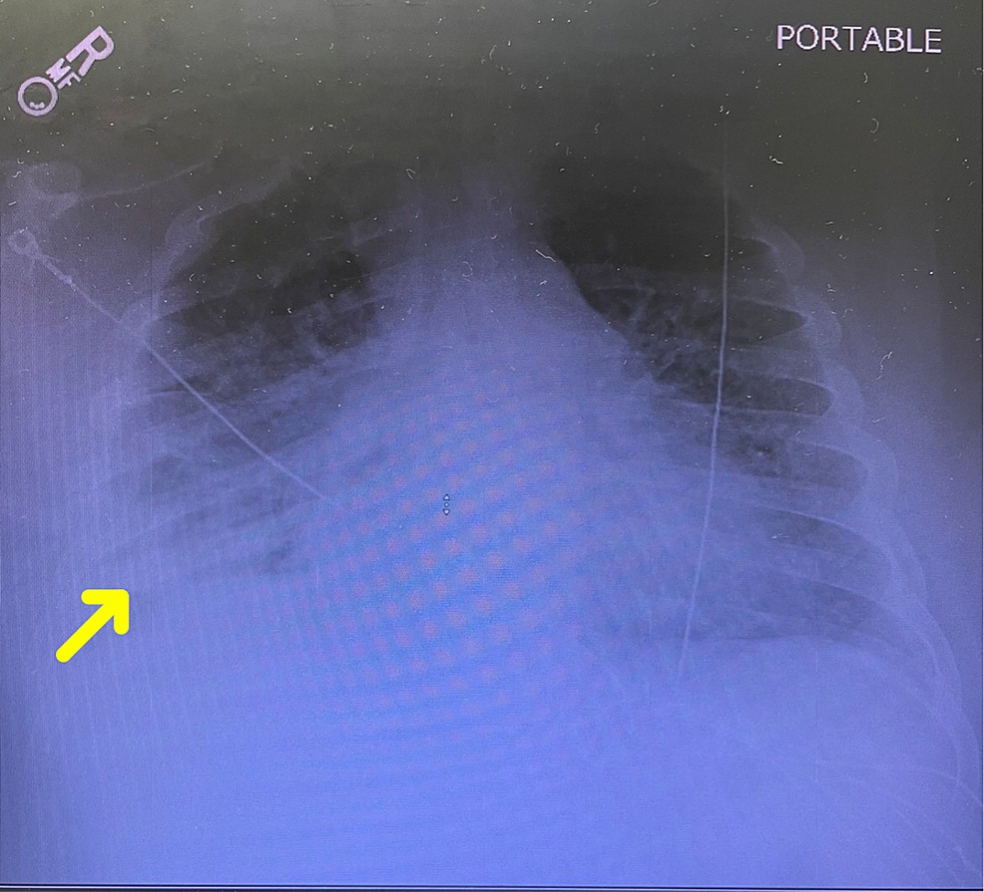
Follow up chest radiograph from day of discharge revealing significantly improved findings from prior imaging studies (yellow arrow)

In the following days, this patient suffered an episode of acute blood loss anemia secondary to a bleed from a Dieulafoy lesion, which resolved after a clipping procedure performed by gastroenterology. The increased propensity for bleeding was attributed secondary to a consumptive coagulopathy from the heparin drip used while this patient was maintained on ECMO. Due to the patient’s persistent leukocytosis and worsening atelectasis noted upon imaging, it was decided that this patient would return to the operating room to undergo a right posterolateral thoracotomy and right lung decortication procedure. The procedure was successful in removing the friable and purulent material secondary to the necrotizing pneumonia. Over the following two weeks, this patient made a significant improvement in his cardiopulmonary function. The lower pressures utilized in the lung containing the BPF via the DLT allowed the BPF to heal, while allowing for adequate oxygenation at higher pressures in the contralateral lung. This patient was successfully extubated to bilevel positive airway pressure, alternating with the use of supplemental oxygen provided through a nasal cannula. Incentive spirometry was also utilized in the recovery of the pulmonary system. Imaging of the chest revealed resolution of the empyema and improvement of the atelectasis (Figure 5). The leukocytosis also resolved, and the ECMO device was removed. The patient was then discharged to a rehabilitation facility for further recovery and scheduled for follow up with the cardiothoracic surgeon for evaluation of surgical incisions. 

## Discussion

Conventional mechanical ventilation with a single lumen ET tube allows for both lungs to be inflated and deflated in synchrony. In certain circumstances, lung isolation techniques, including OLV, are standard methods used to improve surgical field exposure or to anatomically isolate one lung from a pathologic process of the other lung [1]. Some specific indications to use lung isolation to avoid cross-contamination from one lung to the other includes pulmonary hemorrhage, purulent pulmonary secretions, whole lung lavage for pulmonary alveolar proteinosis [1]. Lung isolation ventilation techniques are also used to decrease pressure or air flow on the specified side of pathology; such as in our reported case of a BPF [1]. There are a few contraindications to OLV, such as dependence on bilateral mechanical ventilation and patients with severe pulmonary disease or prior pulmonary resection; in which case, selective lobar blockage may be an option [1]. The insertion of a DLT may also not be indicated in a patient with an intraluminal airway mass that limits access to the bronchial tree and could possibly cause complete airway obstruction subsequent to placement of the DLT [1]. In regards to our patient, conventional ventilation of both lungs was first initiated. This patient remained acidotic and hypercarbic while on mechanical ventilation, and an unusual persistent air leak was noted. It was decided to then exchange the single lumen ET tube for a DLT in order to ventilate the lung containing the empyema at lower pressures while ventilating the contralateral lung at higher pressures in order to maintain adequate oxygenation saturation.

In a brief review of the types of lung isolation, there are three types; left or right DLT, bronchial blocker placement via a single lumen ET tube, and the placement of a single lumen ET tube into a mainstem bronchus [2]. We will further discuss the use of a DLT for OLV, as employed in our patient, which allows for lung isolation, intermittent suctioning of either lung, and selective ventilation of either lung [2]. In a quick comparison of the use of DLT versus bronchial blockers, it was found that bronchial blockers require more repositioning during surgery via fiberoptic bronchoscopy (FOB) [2]. In addition to this finding, a recent meta-analysis of 39 trials found that DLT were positioned 51 seconds quicker on average and more likely to be positioned correctly (odds-ratio: 2.70, 95% CI 1.18-6.18) [2]. The DLT is fashioned with two distinct tracheal and bronchial lumens [2]. The DLT consists of two tubes next to each other; the shorter tube has a larger volume and the lower pressure tracheal cuff, giving rise to the tracheal lumen and is positioned above the carina [2]. The second tube is the smaller volume, a high-pressure bronchial cuff that is placed below the carina but before the branches of any lobar bronchi [2]. There are specified left or right DLTs, however, it is more common to use a left DLT because the length of the left mainstem bronchus is longer than compared to the right mainstem bronchus (measuring an average of 4.9 cm in men and 4.4 cm in women) and allows for more consistently correct placement [2]. Although there are no direct guidelines on how to select the size of the DLT, it is suggested to use the patient’s sex and height [2]. The correct DLT size is confirmed when an air leak is appreciated when the bronchial cuff is deflated but becomes airtight when the bronchial cuff is inflated with an approximate volume of 2-3 mL [2]. The size of the DLT is crucial because the insertion of oversized DLT could lead to trauma or rupture of the trachea or bronchus, and the insertion of a DLT that is too small could result in insufficient lung isolation or lead to obstructive airway resistance [2]. The process of inserting a left DLT begins with passing the endobronchial tip through the vocal cords under direct laryngoscopy, removing the stylet, and then rotating the DLT 90 degrees to the left as the tube is being advanced [2]. In a review of a recent study, it was found that turning and tilting the patient’s head to the right while the DLT is being advanced decreased incidences of the insertion of a left-sided DLT into the right mainstem bronchus in 92% of cases [2]. The gold standard for confirming the position of the DLT is through FOB [2]. Correct placement of the DLT is essential to successful lung isolation, as deep placement of the DLT directly into the bronchus could lead to obstruction of the upper lobe of the lung [2]. For this reason, it is important to place a DLT with the assistance of FOB, as recent studies have shown that 24% to 39% of insertions of left DLT without FOB confirmation resulted in endobronchial intubation of the right mainstem bronchus [2]. It is also important to note that the most common cause of intraoperative hypoxemia during OLV is the malpositioning of the DLT [2]. In a review of 1170 procedures, 1166 of which utilized left DLTs), hypoxemia occurred approximately 3% of the time during OLV, with incorrect positioning of the DLT accounting for greater than 60% of those cases [2].

It is also necessary to discuss the changes in physiology that occur during the differential ventilation of two lungs. Ventilation and perfusion are matched based on their anatomical basis during normal ventilation, as dependent areas of the lungs receive greater blood flow and greater ventilation due to the gravitational effects on compliance [1]. However, when OLV is initiated, one lung is not ventilated entirely, which in theory would create a right-to-left shunt of approximately 50% with resultant hypoxemia if blood flow was not changed, but this does not occur [1]. The actual fraction of shunting that occurs is only about 20% to 30% because of three main reasons: manipulation during surgery obstructs vascular flow to the lung not being ventilated, positioning of the patient laterally leads to the increase in perfusion of the ventilated lung due to gravity, and hypoxic pulmonary vasoconstriction (HPV) [1]. HPV is a mechanism of auto regulation that is initiated in response to an alveolar oxygen partial pressure (PAO2) of less than 100 mmHg [1]. Through vasoconstriction of the pulmonary vessels, HPV redirects blood flow to more ventilated segments of the lung in order for more appropriate ventilation/perfusion matching [1]. In OLV, HPV reduces the blood flow through the non-ventilated lung during OLV by approximately 40% to 50% and mediates the degree of hypoxemia that would otherwise occur [1].

In relation to our patient, who presented with ARDS, conventional mechanical ventilation was insufficient in maintaining adequate cardiopulmonary function due to the significant injury from the empyema. Some of the lung-protective strategies that were initially employed for this patient included low tidal volumes (4 to 6 mL/kg), adjustment of respiratory rate to maintain end-tidal CO2 between 35-45 mmHg, PEEP (5 to 10 cmH2O), plateau inspiratory pressures <30 cmH2O, and permissive hypercapnia, but these interventions were not effective in adequately oxygenating this patient [3]. Subsequent to the evacuation of the empyema and irrigation of the chest cavity, this patient’s pulmonary status continued to deteriorate and a persistent air leak was noted from the chest tubes placed during the procedure. It was then decided to exchange the single lumen ET tube for a left DLT for OLV to further protect the contralateral lung not containing the empyema. The use of a left DLT for OLV improved this patient’s oxygenation status and facilitated the full right lung decortication procedure performed a few days later. OLV also allowed for the cardiothoracic surgeons to have appropriate surgical exposure to further remove the infected lung tissue and identify the source of the persistent air leak: a BPF. The use of OLV was vital to this patient’s postoperative course, as the ability to ventilate the affected lung at lower tidal volumes and pressures allowed for the BPF to heal. Through the clinicians’ utilization of lung protective strategies, a deep understanding of lung physiology, and the application of lung isolation strategies, this patient with an estimated mortality rate of 50% was able to be stabilized over a 33-day hospitalization for discharge to a rehabilitation facility.

## Conclusions

OLV is an infrequently used lung isolation technique typically indicated to improve surgical exposure or isolate the contralateral lung from any pathological processes. Before inserting a DLT for OLV, it is essential to be familiar with the tracheobronchial anatomy and visualize and confirm the placement of the DLT via fiberoptic bronchoscopy. Left DLTs are more commonly used than right DLTs due to the longer length of the left mainstem bronchus, which allows for more consistent placement and less chance of obstructing the bronchus of the upper lobe. The physiological changes that occur during OLV are also important for the clinician to understand, as hypoxic vasoconstriction and lateral positioning during surgery reduce the theoretical shunt of 50% to realistically about 20%-30%. These physiological changes improve ventilation-perfusion matching and help optimize oxygenation in a compromised state. In this unique case of necrotizing pneumonia complicated by a BPF, OLV via a left DLT proved crucial in the recovery of this patient by preserving cardiopulmonary function before and after a lung decortication procedure and ultimately to a favorable prognosis.
